# Stimulation artifact correction method for estimation of early cortico-cortical evoked potentials

**DOI:** 10.1016/j.jneumeth.2016.03.002

**Published:** 2016-05-01

**Authors:** Lena Trebaul, David Rudrauf, Anne-Sophie Job, Mihai Dragos Mălîia, Irina Popa, Andrei Barborica, Lorella Minotti, Ioana Mîndruţă, Philippe Kahane, Olivier David

**Affiliations:** a Univ. Grenoble Alpes, Grenoble Institut des Neurosciences, GIN, F-38000 Grenoble, France; b Inserm, U1216, F-38000 Grenoble, France; c Laboratoire de Neurophysiopathologie de l’Epilepsie, Centre Hospitalier Universitaire Grenoble-Alpes, Grenoble, France; d Neurology Department, University Emergency Hospital, Bucharest, Romania; e Physics Department, University of Bucharest, Bucharest, Romania; f FHC Inc, Bowdoin, ME, USA; h Neurology Department, Carol Davila University of Medicine and Pharmacy, Bucharest, Romania

**Keywords:** Intracranial EEG, Cortico-cortical evoked potentials, Electrical cortical stimulation, Artifact removal, Effective connectivity, CCEP, cortico-cortical evoked potentials, DES, direct electrical stimulation, ECoG, electrocorticography, SEEG, stereo-electroencephalography

## Abstract

**Background:**

Effective connectivity can be explored using direct electrical stimulations in patients suffering from drug-resistant focal epilepsies and investigated with intracranial electrodes. Responses to brief electrical pulses mimic the physiological propagation of signals and manifest as cortico-cortical evoked potentials (CCEP). The first CCEP component is believed to reflect direct connectivity with the stimulated region but the stimulation artifact, a sharp deflection occurring during a few milliseconds, frequently contaminates it.

**New method:**

In order to recover the characteristics of early CCEP responses, we developed an artifact correction method based on electrical modeling of the electrode–tissue interface. The biophysically motivated artifact templates are then regressed out of the recorded data as in any classical template-matching removal artifact methods.

**Results:**

Our approach is able to make the distinction between the physiological responses time-locked to the stimulation pulses and the non-physiological component. We tested the correction on simulated CCEP data in order to quantify its efficiency for different stimulation and recording parameters. We demonstrated the efficiency of the new correction method on simulations of single trial recordings for early responses contaminated with the stimulation artifact. The results highlight the importance of sampling frequency for an accurate analysis of CCEP. We then applied the approach to experimental data.

**Comparison with existing method:**

The model-based template removal was compared to a correction based on the subtraction of the averaged artifact.

**Conclusions:**

This new correction method of stimulation artifact will enable investigators to better analyze early CCEP components and infer direct effective connectivity in future CCEP studies.

## Highlights

• New method for stimulation artifact removal from cortico-cortical evoked potentials.
• The method is based on electrical modeling of tissue-electrode interface.
• It allows disambiguation of time-locked physiological responses and artifacts.
• Method validation is based on synthetic and experimental data.

## 1 Introduction

Brain processing relies on interactions between systems and thus on anatomical connectivity and local dynamics. In neuroimaging and electrophysiology, we often estimate theses interactions using purely observational measures of “functional connectivity”, i.e. the correlation of activity between brain regions, or using other more sophisticated statistical estimators of interactions (e.g. Granger Causality). Investigators generally rely on non-invasive neuroimaging techniques, ranging from functional magnetic resonance imaging (Marrelec et al., 2006; Lang et al., 2012) to magnetoencephalography/electroencephalography (David et al., 2006). Unfortunately, none of these approaches can reliably quantify the direction and chronometry of the interactions. New approaches are warranted that would enable investigators to measure more directly effective connectivity, i.e. the average weight or gain of transmission between regions across anatomical pathways, with acceptable spatial resolution and adequate temporal resolutions, based on invasive techniques using intracranial electrodes (David et al., 2013).

Epileptic patients with drug-resistant epilepsy are sometimes implanted for clinical purposes with intracranial electrodes to localize the epileptogenic brain tissue that has to be removed to render the patient seizure-free. Such intracranial studies enable investigators to simultaneously perform stimulation between (adjacent) contacts and recordings over the electrodes to quantify responses, either using electrocorticography (ECoG) (with subdural strips of electrodes along the surface of the parenchyma) or stereo-electroencephalography (SEEG) (with depth electrodes implanted into the parenchyma across the meninges) depending on the cases (Luders, 2008). In some clinical centers, low frequency stimulations are systematically performed (commonly at 1 Hz), mainly in the hope of inducing seizures that resemble spontaneous seizures and identify their sources (David et al., 2008). Each electrical pulse can induce cortico-cortical evoked potentials (CCEP), which reflect the direct or indirect interactions between the stimulated and recording sites. Two major deflections, usually negative and therefore called N1 and N2 (Matsumoto et al., 2004) although they can also be positive, have been reliably reported. Beyond their importance for the basic and clinical study of epileptic networks (David et al., 2010), CCEP have been used to map functional networks, including networks for language (Enatsu et al., 2013; Koubeissi et al., 2012; Matsumoto et al., 2004), motor control (Conner et al., 2011, Enatsu et al., 2013, Kikuchi et al., 2012, Matsumoto et al., 2012, Matsumoto et al., 2006), and intra- and inter-lobar connectivity (Catenoix et al., 2005, Lacruz et al., 2007, Entz et al., 2014, Keller et al., 2014, Keller et al., 2011). The probability of measuring a significant CCEP, the peak amplitude or the latency of the response following stimulation onset have been used as metrics of connectivity.

**Fig. 1:**
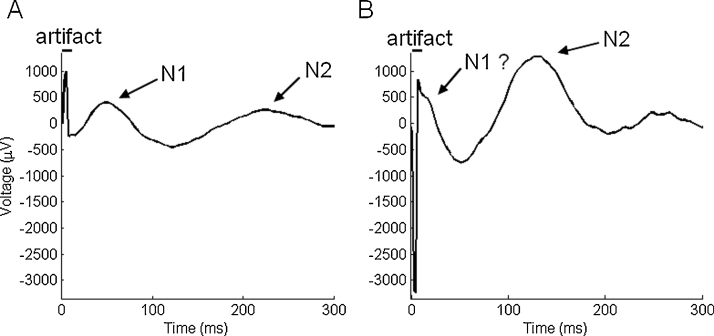
Examples of CCEP responses without (A) and with (B) overlap between the stimulation artifact and the N1 component of the CCEP (recordings from Grenoble, see Section 2.4). Note that the N2 component is usually unaffected by the presence of the artifact.

However, the presence of stimulation artifacts complicates the identification of early peaks when they overlap with the artifact. Current-injection induces a sharp deflection contaminating recordings for at least a few milliseconds, typically 1–6 ms nominally with sometimes longer lasting deflections related to capacitive effects (Fig. 1). Because the use of ECoG requires a stronger current intensity for effective stimulation than SEEG, in ECoG recordings the signal can further be saturated for 5–10 ms (Keller et al., 2014) even when the stimulation only lasts 0.5 ms. Alternating the stimulation polarity is a hardware method that is commonly used in CCEP studies in order to reduce, though not eliminate, the artifact amplitude through averaging (Matsumoto, 2004).

Because electrical stimulations are part of a clinical routine, either to elicit seizures or to map eloquent cortical areas, the low sampling frequency used (often 256, 512 or 1024 Hz) and the potential lack of synchronization between the recording and stimulating systems make the artifact pattern insufficiently sampled and not reproducible between pulses. Therefore, its correction is challenging and this issue has not been fully addressed in the CCEP literature yet. Past and recent CCEP studies generally exclude 5 to 20 ms of signal after stimulation in order to avoid contamination of the analyses by artifact components altogether (Catenoix et al., 2005, Enatsu et al., 2015, Kubota et al., 2013). In that case, the earliest responses occurring during the first 5–20 ms following direct stimulation (Keller et al., 2014) are simply ignored and missed. Finding solutions to reliably quantify these early responses in addition to responses with longer latencies is a mandatory step for being able to precisely demonstrate direct connection pathways, and thus to distinguish direct versus indirect anatomical connections (David et al., 2013). Recently, we proposed a preliminary solution, which corrected the artifact without excluding the concomitant signal, using local cubic spline interpolation applied across a 8 ms window around the stimulation onset (David et al., 2013). We could reconstruct smooth CCEP responses but, in some cases, the solution introduced a bias in the estimates of early CCEP latencies. Here we introduce a novel and more advanced approach capable of better separating electrophysiological signals from the artifact.

Model-driven correction are commonly used for artifact removal in micro- and macro-electrical stimulation and extracellular micro-electrode array recordings, and mainly refers to methods based on the modeling of the stimulation artifact template, which is then subtracted from the measured traces. Often though, the stimulation artifact template is not derived from an explicit biophysical model but from averaging peri-stimulus waveforms or from models including polynomial or exponential functions, and then removed from the original data by subtraction (Erez et al., 2010, Wagenaar and Potter, 2002). In a recent CCEP study (Mouthaan et al., 2016), the approximation of the artifact by an average template was compensated by the use of a Wiener filter, which was applied recursively. The efficiency of a template-based method depends on the pulse-to-pulse variability of the artifact shape, which a low sampling frequency as usual for CCEP data can accentuate. It is thus important to assess the performance of methods based on different recording parameters, notably of sampling frequency. One other important limitation of previous approaches is the inability to distinguish between physiological responses of the tissue that are time-locked to the repetitive stimulation pulses and the purely artifactual component. Using a linear model whose parameters are being tuned for best fit with the data enables allowed us to go around this limitation and to recover the signal during the earliest phases of the stimulation.

In order to constrain the shape of the artifact to a realistic range, we use here a biophysical model of the artifact generation following low frequency (e.g. 1 Hz) direct electrical stimulation (DES). This approach uses the principle of template subtraction based on an electrical model of the electrode–tissue interface and a down-sampling scheme. We generate the set of all possible artifact shapes within a large range of the model parameters and ad hoc sampling frequencies, then identify the best matching template and regress it out from the signal on a single-trial basis. We validate the approach based on simulations and demonstrate its utility on experimental data for different stimulation and recording parameters that encompass most of clinical settings. We focus especially on the preservation of the first CCEP peak shape, the possibility of extracting its latency and of correcting the signals at the single-trial level (e.g. for the study of CCEP plasticity, David et al., 2008).

## 2 Methods

### 2.1 Modeling

#### 2.1.1 Sets of data

We performed simulations in order to compare sets of data with and without stimulation artifacts, with variable amplitudes and latencies of responses. Each set contained 30 channels with a response probability of 50%. The signals were generated at 50 kHz (and then down-sampled at lower frequencies (256, 512, 1064 and 2048 Hz) in order to match experimental data. Each channel *i* contained a signal $D_{a}^{i}$ with a 5 s baseline followed by 40 consecutive stimulations at 1 Hz. 20 sets were analyzed for each parameter (pulse duration and sampling frequency) value. We modeled each stimulation as a sum of three components:

(1) $$\begin{array}{l} D^{i,s}=\text{response}+\text{noise}\\ D_{a}^{i,s}=D^{i,s}+\text{artifact} \end{array}$$

where $D_{a}^{i,s}$ and *D*^*i*,*s*^ are, respectively, the response with and without artifact, corresponding to the stimulation *s* of the channel *i*. $D_{a}^{i,s}$ and *D*^*i*,*s*^ are *n* ∗ *m* matrices, with *n* the number of channels and *m* the number of time samples.

The artifact was modeled as described in the next subsection, with shape, amplitude and polarity change across channels. The first response component was represented by a cosine signal, which amplitude was partially proportional to that of the artifact and the latency of the first peak was distributed between 1 and 40 ms. Its shape variations across successive stimulations were set to be small (modulation of the cosine period of less than 10%) for the same channel and much larger across different channels (modulation of the cosine period up to 66% and modulation of its amplitude up to 100%), including possible polarity change, in order to model standard electrophysiological observations. The term of noise corresponded to a Gaussian distribution of values with zero mean and a variance distributed between 1 and 50 μV. We adjusted the response amplitude so that it exceeds three times the background level in order to estimate easily the peaks latency.

For performance analysis, we extracted the response latency as the latency of the first peak following stimulation onset. We modeled fast responses with a higher frequency (from 50 to 70 Hz) and distributed their peak latencies between 1 and 20 ms, whereas we modeled slow responses with a lower frequency (less than 50 Hz) and distributed between 20 and 40 ms after stimulation onset. When necessary, we truncated fast responses so that their onset always occurred after the stimulation onset.

#### 2.1.2 Artifact

**Fig. 2:**
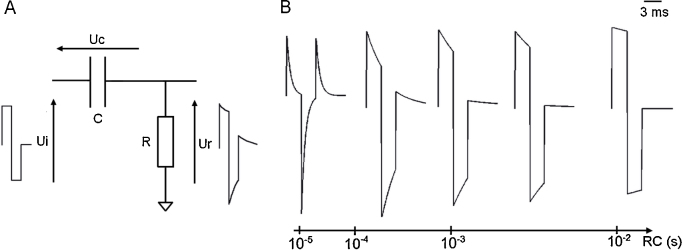
(A) Basic electronic circuit modeling the electrode tissue interface. The capacitance C transformed the square impulse imposed by the stimulator *u_i_* into a damped signal *u_R_* actually going into the brain tissue. (B) The shape of *u_R_* depends on the value of the RC product (see Eq. (2)). Here are represented artifact shapes corresponding to different RC values for a biphasic artifact recorded at 500 kHz for 3 ms pulses.

The artifact was modeled as the output of a RC circuit representing the electrode–tissue interface with a bipolar or monopolar square wave *u*_*i*_ as input (Fig. 2). This circuit provides a simple approximation of the behavior of the electrical potential following current injection between two bipolar contacts through brain tissue. The capacitance *C* summarizes capacitive effects at the electrode/tissue interface. The resistance *R* models passive resistivity of brain tissue. In this model, the recorded signals correspond to the voltage *u*_*R*_ at the level of the resistance. Electrical equations of the model are:

(2) $$\begin{array}{l} \frac{\text{d}u_{c}}{\text{d}t}=\frac{1}{\text{RC}}\left(u_{i}-u_{c}\right)\\ u_{R}=u_{i}-u_{c}\\ \text{artifact}=u_{R} \end{array}$$

We varied the time constant related to RC from 10^−6^ to 10^−2^ s in order to sample the range of possible artifact shapes in the data (Fig. 2B). According to experimental data, the input *u*_*i*_ was a square wave, either monophasic or biphasic. The standard biphasic artifact shape was typically comprised of two peaks followed by a small rebound with exponential decay due to capacitive effects.

### 2.2 Artifact correction

#### 2.2.1 Model-based artifact correction

The correction method was based on the electrical model of artifact described in Section 2.1.2. It required knowing the nominal characteristics of the injected current: its shape (e.g. mono- versus bi-phasic) and duration (e.g. from 1 to 3 ms in our experimental database). We used Eq. (2) of the model of artifact to create a basis of possible artifact shapes that were ad hoc. From our experience, RC values from 10^−6^ to 10^−2^ s were sufficient to cover all the possible shapes encountered in real data. Simulations were performed at 50 kHz before parametric down-sampling aimed at matching experimental sampling frequencies. The different possible shapes induced by down-sampling were generated by shifting the first sample of the high frequency model recursively and by down-sampling the signal for every shift. These different shapes were stored for every artifact template (i.e. for a given RC value) and compared by linear regression to every artifact detected in simulated or empirical data across a time window of 1.2 times the pulses duration, or corresponding to at least 3 samples. We reasoned that the actual responses are less likely to influence the part of the signal containing the artifact pulses than parts corresponding to the slow decay that can easily be mixed up with an early response. For each RC value, we selected the best fit for each one of the stimulations by minimizing the residual power between the regressed signal and the actual signal. The minimal residual power summed on each stimulation run allows the selection of the RC value corresponding to global best fit for each channel. The corresponding fitted artifacts were removed from the data using linear regression. Finally, we removed remaining frequencies above 90 Hz using a low-pass filter.

#### 2.2.2 Average-based artifact correction

We compared our electrical model-driven method with such a simpler method consisting in the subtraction of the average signal around the artifact calculated for every channel as is commonly used to suppress different kinds of artifacts (Erez et al., 2010, Hashimoto et al., 2002). More specifically, the average template was calculated by selecting the signal following stimulation onset for the nominal duration of the expected artifact extended by 1 ms to correct the approximation due to down-sampling, subtracting the value of the first sample (considered as the baseline) and averaging data within this time window across the different stimulations. In the same way as for the other method, we removed remaining frequencies above 90 Hz using a low-pass filter.

### 2.3 Performance metrics

We used two different metrics to evaluate the performance of the correction methods in simulated data for which we knew the ground truth *D*. For each data set *D_c_*, we calculated them for each stimulation *s* of each channel *i* and then averaged them over the stimulations.

The *residual power* (*RPower*) is the ratio between the artifactual power remaining after correction and the response power:

(3) $$RPower^{i}={\text{mean}}_{t}\left(\frac{\text{power}\left(D_{c}^{i,s}-D^{i,s}\right)}{\text{power}\left(D^{i,s}\right)}\right)$$

It was applied from each stimulation onset to 10 ms after, which is the peak latency range considered.

The *latency bias* (*LBias*) compares first peak latencies for the simulated (*l*) and corrected (*l*_*c*_) responses. The first peak latency corresponds to the latency of the first maximum that is at least two times the standard deviation of the signal.

(4) $$LBias^{i}=\left|{\text{mean}}_{t}\left(l_{c}^{i,s}-l^{i,s}\right)\right|$$

where $l_{c}^{i}$ the corrected latency and *l*^*i*^ the latency expected considering the sampling frequency of the channel *i* and the stimulation *s*.

Good performance implies minimizing both *RPower* and *LBias*.

### 2.4 Experimental data

Experimental data were selected from two different clinical centers (Grenoble University Hospital and Bucharest University Emergency Hospital) participating to the research protocol F-TRACT (INSERM IRB 14-140). The patients gave their consent to undergo invasive recordings and 1 Hz stimulation as part of a presurgical evaluation of their drug-resistant epilepsy (Grenoble: ID RCB 2013-A01098-37; Bucharest: Protocol No 2621/03.02.2012). In Grenoble, runs of 40 s stimulations were performed between two contiguous contacts in the grey matter using monophasic 3 ms duration pulses of 3 mA. Signals were recorded at 512 Hz. For more details about the standard clinical procedure, see David et al. (2013). The procedure for data acquisition was similar in Bucharest, using a biphasic pulse of 3 ms and a high sampling rate of 4096 Hz. The full procedure is explained in Donos et al. (2016).

## 3 Results

### 3.1 Modeled data

#### 3.1.1 Example: Correction of successive single pulses

**Fig. 3:**
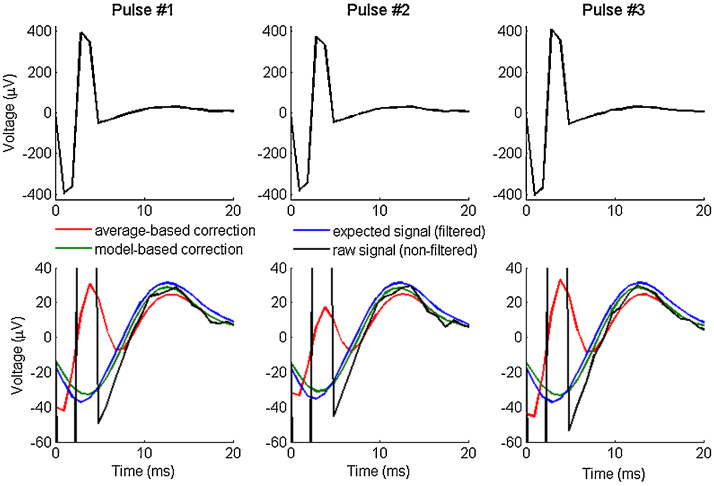
Example of modeled time series corresponding to three consecutive stimulations (the first 30 ms after the stimulations onsets) with a sampling frequency of 1024 Hz and a biphasic artifact of 2 ms pulse duration: (up) the whole signal with the artifact, (down) zoom on the response. The raw signal (black) corresponds to the simulated signal with artifact whereas the expected signal (blue) is the same without the artifact and filtered to removed high frequencies like the two corrected signals (red and green). (For interpretation of the references to color in this figure legend, the reader is referred to the web version of this article.)

Fig. 3 represents an example of a signal with a fast response corrected using the model-based and the average-based method for a sampling frequency of 1024 Hz and a biphasic artifact with 2 ms pulse width. On the signal corrected using the average-based correction, part of the artifact remains and can be confounded with a response after low-pass filtering. With the model-based method, the reconstructed signal exhibits a shape similar to that of the expected signal and is reproducible across successive stimulations. The peak latency extracted from the model-based corrected signal corresponds to the expected one, whereas the average-based method will clearly induce a bias.

#### 3.1.2 Influence of recording and stimulation parameters

We studied the influence of sampling frequency and pulse duration on the modeled data using the performance metrics on 20 data sets for each parameters pair. We focused our analysis on fast responses with a latency peak between 1 and 10 ms (which corresponded to 84 to 89 channels depending on the stimulation parameters). We represented the median and the standard deviation of the performance metrics calculated for each considered channel.

**Fig. 4:**
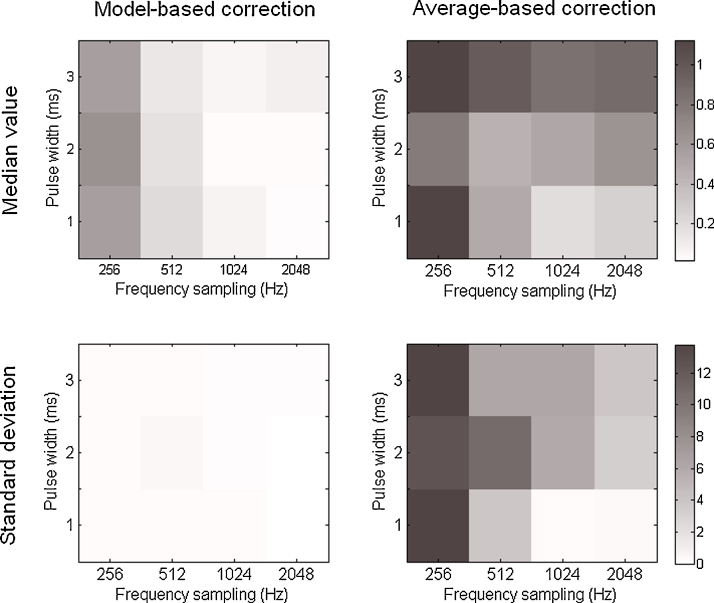
*RPower* results for the model-based (left) and the average-based (right) corrections. On top and bottom rows are represented the median value and the standard deviation obtained for each parameters pair across the analyzed signals.

The *RPower* is represented on Fig. 4 for the model-based and the average-based method. The median *RPower* is lower for the model-based correction method than for the standard average-based one irrespective of the different parameters. It is also higher for low frequency sampling (in particular at 256 Hz) than for higher ones. The pulse duration does not remarkably influence the results for the model-based method whereas the results are worse for larger pulse duration with the average-based method. Values of 1 in the results, as obtained using the average-based method, mean that there is a remaining artifact after correction with a power scale similar to that of the response which will make the peak detection and the response analysis difficult. The low standard deviation of the *RPower* for the model-based method in comparison with the one obtained for the average-based method demonstrate the robustness of the correction.

**Fig. 5:**
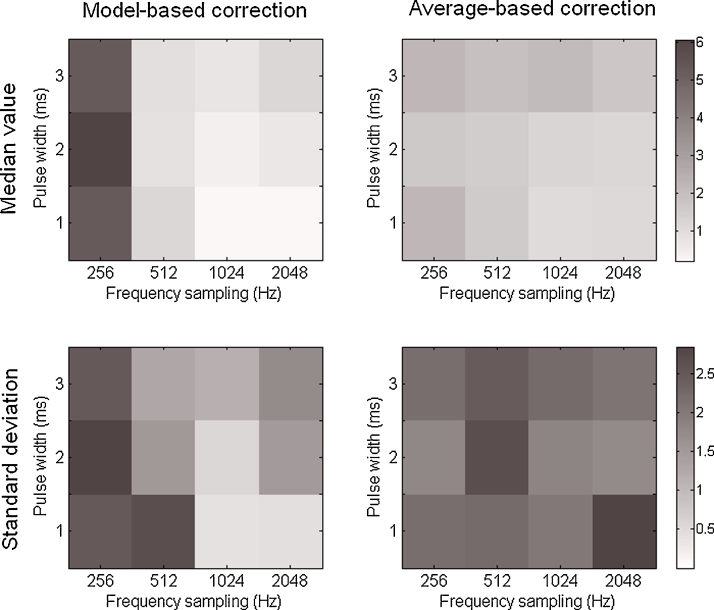
*LBias* (in milliseconds) results for the model-based (left) and the average-based (right) corrections. On top and bottom rows are represented the median value and the standard deviation obtained for each parameters pair across the analyzed signals.

The *LBias* (Fig. 5) indicates with how much precision we can extract the peak latency of fast responses. For the model-based method, the median *LBias* is in general very low (less than 1 ms) except at 256 Hz (around 5 ms). The standard deviations remain quite low, rarely exceeding 2.5 ms for the two methods and remaining around 1 ms for the model-based correction. We must analyze the results obtained for the comparison method in regards to the *RPower* results: the *LBias* measures the first sample above a given threshold so that the response can be confused with the remaining artifact if its amplitude scale is similar (see the example in Section 3.1.1). Therefore, these results on early responses do not really reflect the efficiency of the average-based correction to separate response peaks from the artifact.

### 3.2 Experimental data

**Fig. 6:**
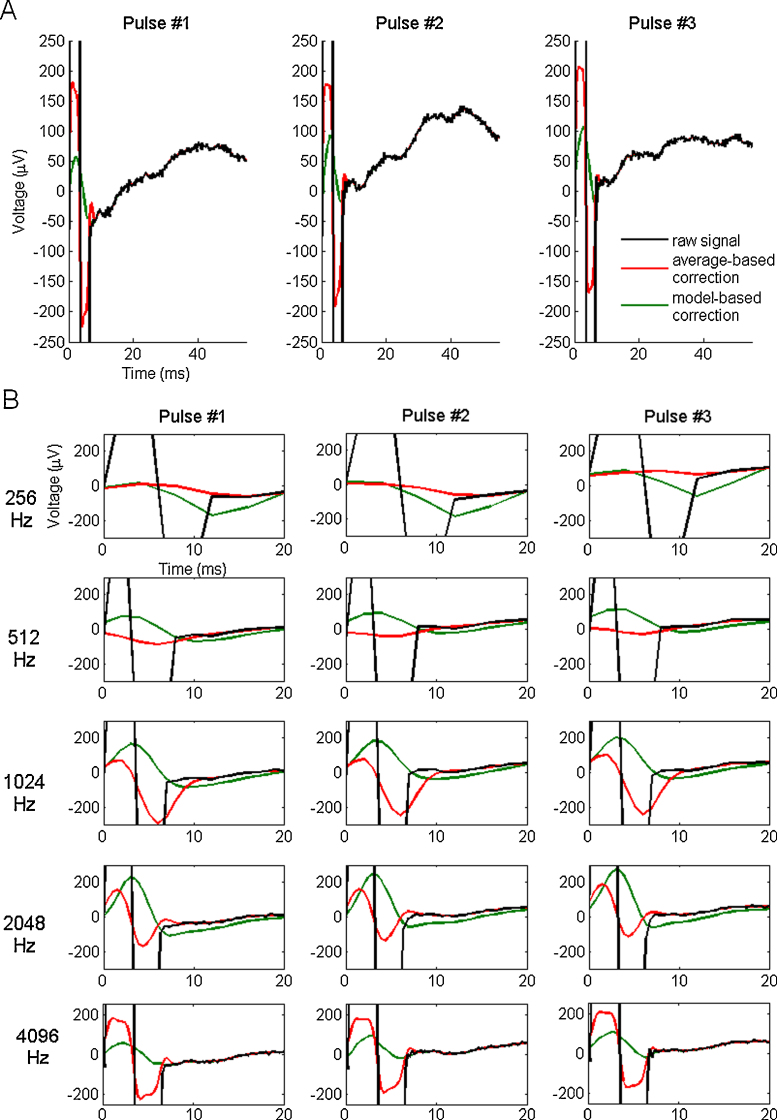
Signals corresponding to three consecutive stimulations (biphasic artifact, pulse width of 3 ms), with the raw signal (black) and the recordings corrected using the model-based method (green) and the average-based method (red), for a time window of 55 ms and a frequency sampling of 4096 Hz (A) and for a smallest time window (20 ms) and different sampling frequencies: 256, 512, 1024, 2048 and 4096 Hz (B). (For interpretation of the references to color in this figure legend, the reader is referred to the web version of this article.)

**Fig. 7:**
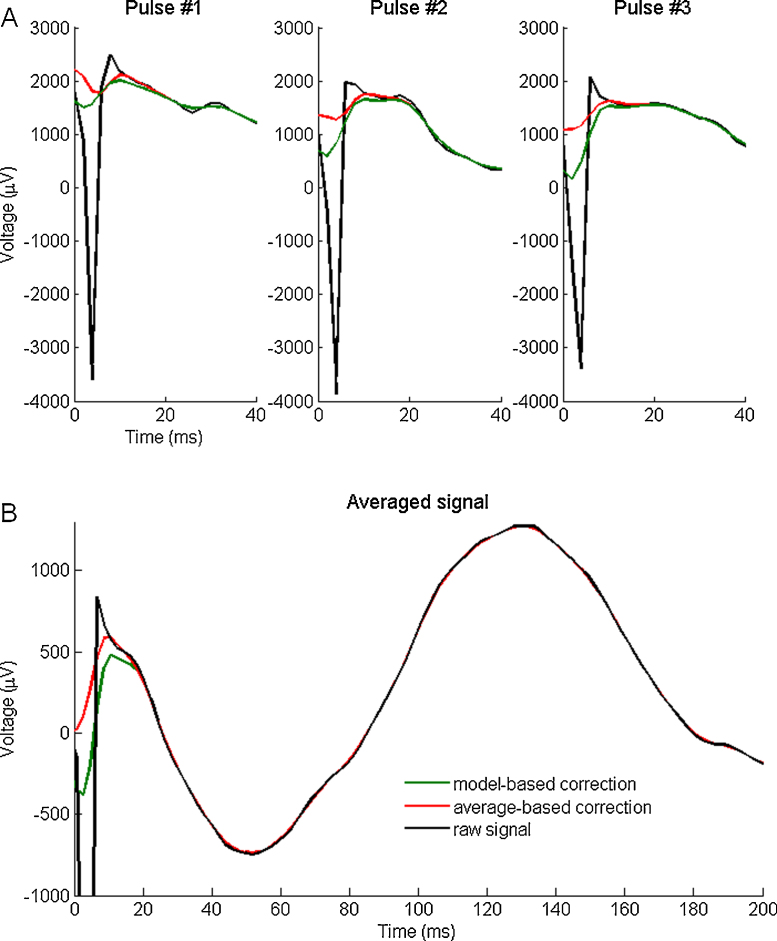
Signals corresponding to three consecutive stimulations (monophasic artifact, pulse width of 3 ms), with the raw signal (black) and the recordings corrected using the model-based method (green) and the average-based method (red) recorded at 512 Hz represented for three consecutive stimulations (A) and averaged over a run of 40 stimulations (B). (For interpretation of the references to color in this figure legend, the reader is referred to the web version of this article.)

We applied the two methods to experimental data, including cases in which early responses were apparent. Fig. 6 shows an example of the correction results on a recording in the paracentral lobule after stimulation in the lingual gyrus with a biphasic artifact with a 3 ms pulse, at the four different sampling frequencies used for the simulations and at the initial sampling frequency (4096 Hz) that is unusual and therefore valuable (Bucharest data). Fig. 7 shows the correction results on a monophasic artifact with a 3 ms pulse recorded at 512 Hz (Grenoble data). Both the recording and the simulated electrodes were in the temporo-parietal junction.

In Fig. 6, we examined the corrections of a 3 ms pulse biphasic artifact for the sampling frequencies matching the ones analyzed for the modeled data. On these recordings, outside the artifact period, only a slow wave could be seen with peak latency around 40 ms (Fig. 6A). The model-driven correction extracted a first positive peak around 3–4 ms with good reproducibility for signals sampled at 512, 1024, 2048 and 4096 Hz (Fig. 6B). The extraction of such deflection did not occur for a sampling frequency of 256 Hz as the frequency of the responses to be extracted in this case comes close to Nyquist frequency. The shape of the first peak that was clearly distinguishable following model-based correction corresponds to a classic sharp deflection corresponding to a fast neuronal response. Because its shape was not biphasic as the stimulation artifact, it is not likely to correspond to artifact residual. This is in contrast with the average-based correction that extracted peaks with a variable shape depending on the sampling frequency and resembling to artifact one, especially at 4096 Hz. These peaks are likely to correspond to artifact residuals as observed with simulated data.

Fig. 7 shows one example of Grenoble data, with a likely early response's shape with peak latency around 10 ms that the artifact partially obscured. Whereas the average-based method seemed to only remove the contaminated part of the signal, an effect that appeared clearly on the averaged signal (Fig. 7B), our correction method adapted the response amplitude based on the capacitive effects that induced a deflection after the pulse for each trial (Fig. 7A). The first early negative peak observed in the averaged signal before the positive peak occurring 20 ms after the pulse may correspond to a response shape already reported in some studies (Terada et al., 2012, Usami et al., 2015). This example shows that, even if the signal contamination by the artifact pulse seems to be small, the first peak characteristics may be affected.

## 4 Discussion

The development of artifact correction solutions for intracranial EEG/DES is critical for methodologies and projects building-upon CCEP, as in the F-TRACT project (David et al., 2013), in order to develop large-scale atlases of connectivity. In this project, a wealth of data is collected across multiple centers around the world that are acquired in clinic routines, generally with different acquisition and stimulation parameters. In this context, one of the central goals for functional mapping is to find solutions enabling investigators to reliably detect and quantify early responses with short latencies, without changing the parameters used for clinical purpose across the centers. For this purpose, we developed a correction method based on a generative model of the artifact in order to better separate the stimulation artifact from the neuronal signal. This approach enables a disambiguation of the repetitive physiological responses from the purely artifactual components. We tested this correction on simulations with different stimulation and recording parameters encompassing all known clinical settings, evaluated with performance coefficients and applied to experimental data. This method allowed us to extract early responses in experimental data and should allow investigators to more reliably analyze, in the majority of cases, early response shapes and first peak latencies at the single trial level, as well as on averaged signals. It is particularly important because those early CCEP components are the most reliable for the inference of direct connectivity with the stimulated site.

First, the validity of the model used to simulate and correct the artifact after down-sampling in the model-driven method is questioned. Then, the effect of the precision of the peak latency extracted after correction that has been quantified on the simulations is analyzed. Finally, we discuss the problem of low sampling frequency and how it can affect recordings and artifact correction.

We modeled the capacitive effects of the brain-electrode interface on the shape of the recorded artifact with an RC circuit. This is of course a simplistic model of what really happens to currents and potentials across electrodes implanted in the brain, with its complex conduction properties. In Merrill et al. (2005) a more sophisticated model has been developed in order to better understand what happens at the level of the electrode–tissue interface. However for the specific use-case of CCEP artifact correction, which will generally be applied on poorly sampled signals, the difference between the RC circuit and a more sophisticated model simulating artifact shape is not expected to be substantial. Moreover, when corrections are to be applied on multicentric data, practical limitations may often render difficult to envision precise modeling and stimulation of specific acquisition systems. For this reason, a simplistic model such as the RC circuit might be a good and convenient trade-off, all the more as the precise modeling of subtle effects of signal modulations related to the specifications of acquisition/stimulation systems might most of the time be ineffective in fitting highly degenerate data that are sampled at a low frequency.

A small peak latency bias induced by the correction has been quantified on the simulations for early responses with a peak latency inferior to 10 ms. For sampling frequencies above 256 Hz, the peak latency estimated after correction is close to the real peak latency with a bias generally between 0 and 2 ms in the worst case scenario. We note that such bias only corresponds to one or few samples for sampling frequencies between 256 and 2048 Hz. Such precision should be sufficient for the practical investigation of connectivity, even between regions that are anatomically close. Nevertheless, future analyses using the correction method will have to acknowledge this (rather small) limitation in the accuracy of latency estimation, of about 1 ms.

A low sampling frequency, such as 256 Hz or below, will not be able to evidence fast events that come close to the Nyquist frequency with sufficient precision. The recordings will not capture a monophasic artifact with a pulse duration that is less than 4 ms or a biphasic one with pulse duration that is less than 2 ms and thus we will not estimate the stimulation onset adequately. The precision of the stimulation onset is particularly important for the model-driven method that fits simulated artifact shapes to data based on the sample corresponding to the onset of the artifact detected in the data. The shape of the fast early responses will also be more difficult to reconstruct with only a few data samples to represent it. Investigators have to deal with data acquired at such low sampling frequencies due to yet irreducible clinical constraints, and try to develop methods that can adapt to them, but we will surely miss some information as a result. Nevertheless, based on these results, we recommend that clinical centers capable of acquiring and storing data at higher sampling frequency adopt a sampling frequency of at least 512 Hz if they wish to be able to study these data with advanced computational methods. This does not change anything in terms of clinical practice, since stimulation parameters as utilized in routine do not have to be modified—which would represent an ethical issue. The only constraint is to increase sampling frequency capacities, which obviously raises the issue of costs.

## 5 Conclusion

We developed an artifact correction method for the study of CCEP responses, based on a simple electrode–tissue model with a higher performance than a traditional method such as average subtraction. This method separates the neuronal signal from the stimulation artifact and preserves the shape of the responses at the single-trial level. With the method proposed in this study, we can reliably study CCEP responses, even early ones, in particular over large quantity of data acquired with different settings, in a manner that will allow investigators to quantify functional connectivity and estimate effective connectivity at the large-scale.
